# The diagnostic value of endoscopic ultrasound for esophageal subepithelial lesions: A review

**DOI:** 10.1097/MD.0000000000040419

**Published:** 2024-11-15

**Authors:** Wanwen Li, Mengqi Shao, Shichen Hu, Shenglong Xie, Bin He

**Affiliations:** a Department of Thoracic Surgery, Sichuan Provincial People’s Hospital, School of Medicine, University of Electronic Science and Technology of China, Chengdu, China; b Department of Thoracic Surgery, The Second Xiangya Hospital of Central South University, Changsha, China.

**Keywords:** artificial intelligence, diagnosis, endoscopic ultrasound, esophageal subepithelial lesions, fine needle aspiration

## Abstract

Esophageal subepithelial lesions (ESELs) encompass a variety of diseases, including leiomyoma, granular cell tumors, hemangioma, lipoma, stromal tumors, leiomyosarcoma, schwannoma, neuroendocrine tumors and more. These lesions often present asymptomatically, leading to a generally low clinical diagnosis rate. Common imaging techniques for diagnosing ESELs include conventional endoscopy, spiral computed tomography, and endoscopic ultrasound (EUS). Among these, EUS is currently regarded as one of the most accurate methods for diagnosing ESELs. In recent years, EUS has increasingly been combined with advanced technologies such as artificial intelligence, submucosal saline injection, high-frequency impedance measurement, and enhanced imaging to improve diagnostic accuracy and reduce missed diagnoses. This article reviews the application and recent advancements of EUS in diagnosing esophageal submucosal lesions.

## 1. Introduction

**Figure 1.**
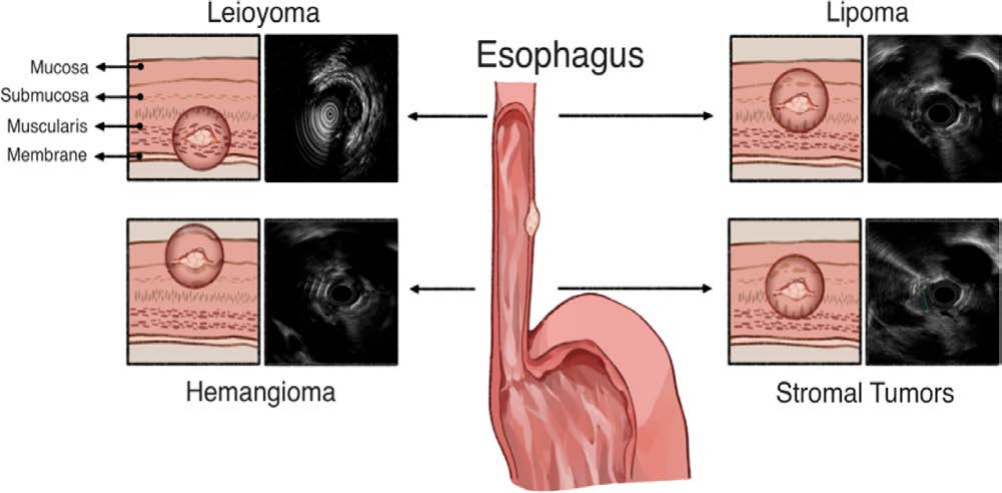
The origin and the characteristic appearance of 4 common esophageal subepithelial lesions (including leiomyoma, granular cell tumor, lipoma, and hemangioma) on EUS. EUS = endoscopic ultrasound.

Esophageal subepithelial lesions (ESELs) encompass a range of diseases. Especially <2 cm in diameter, ESELs often remain asymptomatic and are usually discovered incidentally during medical examinations or endoscopies. It is reported that ESELs are found in approximately 1 out of every 300 patients undergoing routine endoscopy.^[1]^ The most common benign histological types of ESELs include leiomyomas, granular cell tumors (GCTs), hemangiomas, lipomas, and esophageal gastrointestinal stromal tumors (GISTs), with leiomyomas being the most prevalent, accounting for approximately 90% of benign tumors.^[2,3]^ The typical layer of origin of ESELs are illustrated in Figure 1.

ESELs also encompass several malignant lesions, such as lymphatic sarcoma, fibrosarcoma, hematopoietic system tumors, melanoma of neural crest origin, and esophageal carcinoma from foregut amine precursor uptake and decarboxylation lineage cells.^[4,5]^ Accurate diagnosis and differential diagnosis of ESELs are crucial for determining the appropriate treatment. Therefore, improving diagnostic accuracy and the ability to correctly differentiate between types of ESELs has become essential.

Endoscopic ultrasound (EUS) plays a crucial role in diagnosing gastrointestinal (GI) lesions.^[6,7]^ In recent years, EUS has been extensively utilized in diagnosing and treating liver cancer,^[8]^ gallbladder cancer,^[9]^ pancreatic cancer,^[10]^ and upper GI cancer.^[11]^ By providing high-resolution and real-time imaging of luminal and wall structures, EUS allows for the assessment of the lesion’s origin, echo level, and internal echo pattern. This capability enables more accurate qualitative diagnosis of ESELs and T-staging of esophageal malignancies.^[12]^

During an EUS, the wall of the GI tract is divided into 5 layers: a thin hyperechoic layer (superficial mucosa); a hypoechoic layer (mucosal muscularis); a hyperechoic layer (submucosa); a thick hypoechoic layer (muscular layer); an outermost hyperechoic layer (outer membrane). Due to the malignant potential of some ESELs, regular follow-up with EUS may be necessary even for lesions smaller than 30 mm, as they often do not produce obvious clinical symptoms.^[13]^ EUS is considered the “gold standard” for evaluating the location and depth of infiltration of localized ESELs.^[14]^ Compared to spiral computed tomography (CT), EUS has a higher diagnostic rate for subepithelial lesions in the digestive tract, exceeding approximately 15%.^[15]^ EUS has distinctive advantages in determining the size, nature, infiltration depth, and surrounding microstructure of lesions.^[16]^ Common EUS techniques include: linear endoscopy; radial endoscopy; fine needle aspiration (FNA) and fine needle biopsy (FNB). Table 1 lists the characteristics of these techniques.

**Table 1:** Common EUS techniques.

EUS technologies	Feature	Clinical applications
Linear echo endoscopy	Sagittal plane generates images More limited field of view Inability to fully observe the wall of the tube	Widely used in tissue sampling
Radial endoscopy	Provides a 360-degree view of the image Comprehensive observation of esophageal lumen and inner wall structures	Widely used in the diagnosis and T-staging of cavity organ tumors
Fine-needle aspiration (FNA) and Fine-needle biopsy (FNB)	Obtain a small amount of tissue and cells from the lesion Definitive pathologic diagnosis	Often used in the pathologic diagnosis of tumors

EUS = endoscopic ultrasound.

## 2. Diagnosis of ESELs by EUS

EUS can detect suspected intraesophageal and paraesophageal masses, as well as regional lymph nodes, it also enables precise fine-needle aspiration and biopsy (FNA/FNB).^[17,18]^ Both of the American Gastroenterological Association and the European Society of Gastrointestinal Endoscopy recommend EUS as the most accurate methods for diagnosing ESELs.^[1,11]^ The characteristics and applications of common ESELs under EUS have been listed in Table 2.^[11]^ Despite EUS’s unique advantages in the diagnosis of ESELs, the diagnosis of morphological characteristics and sources still partly relies on the experience of endoscopists. EUS combined with FNA/biopsy can further clarify the pathological diagnosis, reduce subjectivity, and enhance the reliability of diagnosis. The indication for EUS-FNA in ESELs is for lesions originating from the fourth layer, exhibiting malignant signs such as heteroplasm, nodules, ulcers, and anechoic zones are present, as well as abnormal types of ESELs.^[12]^ By obtaining appropriate cytologic samples, EUS-FNA can provide more accurate pathological diagnosis and differential diagnosis of ESELs including leiomyoma and schwannoma, GIST, cyst, and rare diseases such as neuroendocrine tumor (NET), glomus tumor and GCT.^[19]^ EUS-FNB or mucosal incision assisted biopsy are also recommended as routine methods for the examination of subepithelial lesions in the digestive tract larger than 2 mm in diameter.^[11,20]^ One study has found that mucosal incision assisted biopsy was no significantly different in accuracy from EUS-FNA in practice, while the operation time was longer than that of FNA.^[21]^ However, the sensitivity of EUS-FNB was 28% higher than that of EUS-FNA (79.41% and 51.92%). The accuracy was increased by about 11% (88.03% and 77.19%).^[22]^

**Table 2:** Characterization and application of common esophageal subepithelial lesions under EUS.

Common esophageal subepithelial lesions	Main sites of origin	Echoic structure	Typical characteristic
Leiomyoma	60% to 80% Layer 4, some layer 2	Slightly hypo-echoic	Clear boundary
Granular cell tumors	Layer 4	Hypo-echoic, echoes slightly above the muscular layer	Homogeneous lesions, yellowish-white
Hemangioma	Layer 2, 3	Hypo-echoic	Clear boundary
Lipoma	Layer 3	Hyper-echoic	Clear boundary, pale yellow
GIST	Layer 2, 4	Hypo-echoic	Heterogeneous mass with a cyst cavity
Leiomyosarcoma	Layer 4	Homogenous echoic	Irregular tumor margins with cystic spaces
Schwannoma	Layer 4	Hypoechoic	Clear boundary
Neuroendocrine tumors	Layer 2, 3	Any echoic	Clear boundary

EUS = endoscopic ultrasound.

### 2.1. Esophageal leiomyoma

Esophageal leiomyoma is the most common subepithelial lesions of the esophagus, most of which originates from the muscularis mucosa (about 60–80%) and typically occurs in the lower 1/3 of the esophagus.^[23]^ On EUS, most esophageal leiomyoma are homogeneous, endogenous, and spherical.^[24]^ The primary imaging examinations for diagnosing esophageal leiomyoma include spiral CT, conventional endoscopy and EUS.^[25]^ Although ordinary spiral CT and conventional endoscopy can locate esophageal leiomyoma and differentiate them from esophageal polyps and esophageal cancers, they are less effective at distinguishing them from other benign lesions such as GCTs, lipomas, and hemangiomas.^[26]^ EUS not only identifies small ESELs, but also plays a role in the differential diagnosis of esophageal leiomyoma by accessing the origin, size, location and relationship between the lesions and surrounding organs. Preoperative diagnosis by EUS has shown a diagnostic accuracy of 88.6% for esophageal leiomyoma, which is significantly higher than that of other imaging examinations.^[27]^ In a retrospective analysis of 1885 cases diagnosed with esophageal leiomyoma via EUS, the preoperative diagnostic accuracy was found to be 86.2%, outperforming conventional endoscopy and CT.^[28]^ These findings suggest that EUS is the preferred imaging modality for esophageal leiomyoma. However, due to the slow growth and progression of esophageal leiomyoma, long-term EUS follow-up may not be necessary for asymptomatic, small-diameter esophageal leiomyoma cases.^[29]^

However, EUS is not specific in distinguishing intramural masses with high of cystic contents density, especially leiomyomas and extramucosal cysts. When the nature of the lesion is not distinctive, ordinary examination is likely to lead to missed diagnosis and misdiagnosis. Combining EUS with FNA can enhance diagnostic accuracy by obtaining cellular and tissue samples for pathological analysis with minimal trauma.^[30]^ In such case, the application of EUS can effectively classify the levels of esophageal lesions, diagnose the lesions of different origins separately, and facilitate endoscopic submucosal dissection (ESD) to achieve the therapeutic purpose if necessary.^[25]^ In addition, when esophageal leiomyoma and esophageal low-grade intraepithelial neoplasia coexist, EUS can confirm the tumor’s origin from the mucosal muscular layer, enabling endoscopic resection of smaller tumors, and reducing the risk of missed diagnosis and over-examination to a certain extent.^[31]^

### 2.2. Esophageal GCTs

GCT is a relatively common ESELs, with an incidence second only to that of esophageal leiomyomas.^[32]^ With the development of imaging, the detection rate of GCTs is gradually increasing. GCTs typically originate from the fourth layer, often occurring in the lower 1/3 of the esophagus. Under conventional endoscopy, GCTs appear as yellowish-white masses with clear boundaries and a slightly tough quality.^[33]^ On EUS, GCTs showed hypoechoic changes, uniform internal echo, well-defined masses with most of the myometrium and adventitia intact.^[12,15]^ For GCTs, EUS is a more accurate imaging modality than white light endoscopic. One study showed that the difference in diagnostic rates between the same endoscopists with EUS and white light endoscopic was nearly 35%.^[34]^

Similar to leiomyoma, GCTs mainly appears as solid lesions with clear boundaries and uniform density under conventional endoscopy and spiral CT, which is easy to be confused in clinical practice.^[35]^ However, unlike leiomyomas, GCTs are benign tumor with malignant potential, necessitating slightly different treatment plans.^[36]^ Therefore, distinguishing between the 2 is crucial. In EUS, more than 90% of GCTs exhibited significantly hyperechoic than the normal muscular layer, while leiomyoma’s echoes were similar to the normal muscular layer.^[15]^ In addition, compared with normal esophageal epithelial cells, the hyperechoic epithelial lining of most GCTs are blurred, while those of leiomyomas are generally clear (26.7% vs 100%, respectively).^[15,37]^ This typical difference makes EUS an important imaging examination in differentiating between the 2.

### 2.3. Esophageal hemangioma (EH)

EH is a relatively rare benign tumor among ESELs, and is commonly found in the lower esophagus.^[38]^ EH usually originates in mucosa or submucosa and presents as well-defined hypoechoic mass under EUS.^[11]^ Smaller diameter EH usually lack specific clinical manifestations, but EH with large diameter may cause concealed dysphagia and hemorrhagic anemia, similar to the clinical presentations of esophageal varices, leading to potential misdiagnosis.^[39]^

EUS plays an important role in the differential diagnosis of EH and varices. On EUS, EH is shown as well-defined hypoechoic masses, whereas esophageal varices appear continuous and intact luminal tracts.^[27]^ This significant difference can make differential diagnosis clear. Furthermore, for EH larger than 2 cm in diameter, ESD is an effective treatment with few long-term complications and EUS can help the treatment of ESD. Additionally, endoscopic embolization with an esophageal coil guided by EUS can achieve good long-term results with minimal incisions.^[40]^

### 2.4. Esophageal lipoma

Esophageal lipoma is a relatively rare subepithelial lesions of the esophagus. The main symptoms include dysphagia (64.2%), polyp reflux (32.4%), and globular sensation (22.7%).^[41]^ More than 85% of esophageal lipoma occur in the cervical esophagus, and progress into the esophageal lumen.^[41]^ Esophageal lipomas present as typical masses with hyperechoic and clear boundaries on EUS, and the diagnosis is relatively clear under EUS, so EUS-FNA is not recommended as a routine procedure.^[12,42]^ However, some researchers have found that the origin and characteristics of esophageal adenocarcinoma are very similar to those of lipoma on EUS.^[12,43]^ Contrary to the benign results of EUS and submucosal biopsy, pathological examination results in some cases has indicated malignant tumor, highlighting EUS still has some loopholes and misdiagnosis rate in the diagnosis of esophageal lipoma.^[41]^

### 2.5. Esophageal stromal tumors

GISTs are potentially malignant tumors that mainly originate in the stomach and small intestine, while only 5% originating in the esophagus.^[44]^ As a relatively rare ESELs, the diagnostic rate of esophageal stromal tumors by conventional imaging is low. Previously, CT scans and positive staining for CD117 (C-Kit), CD34, and/or DOG-1 were the most commonly used diagnostic indicators.^[45]^ But EUS plays a crucial role in diagnosing esophageal stromal tumors. The European Society of Gastrointestinal Endoscopy suggests that the accuracy of EUS in diagnosing GIST is 77% to 89%.^[11]^ On EUS, high-risk esophageal stromal tumors do not present consistently with low-risk ones. EUS-FNA is a rapid and economical imaging method for GI tumors, and the consistency of cytological specimen results with postoperative histology is close to 100%.^[46]^ However, due to the small sample size, the accuracy of EUS-FNA results is limited, and it is prone to false negatives. Compared with EUS-FNA, EUS-FNB can gain more tissue samples, for the diagnosis of esophageal mesenchymal tumors, its accuracy may be superior to EUS-FNA.^[47]^ However, invasive examination increases the risk of bleeding and scarring, which may lead to esophageal stenosis.^[48]^

In recent years, contrast-enhanced EUS (CH-EUS) has been gradually applied to the diagnosis of esophageal stromal lesions. This technique combines ultrasonographic endoscopy with enhanced imaging to diagnose esophageal lesions by detecting microvascular enhancement. CH-EUS has considerable advantages in diagnosing esophageal stromal tumors, clarification of malignant potential and differential diagnosis. On CH-EUS, esophageal stromal tumors often appear as hyper-enhancing lesions, whereas leiomyoma and lipoma are usually hypo-enhancing lesions. This clear distinction improves the diagnostic accuracy of esophageal stromal tumors and differentiates them from other benign esophageal diseases. In 2012, a study performed CH-EUS on 17 suspected esophageal/gastric lesions, revealing that 8 high-enhancing lesions were esophageal stromal tumors, and 9 low-enhancing lesions were 4 lipomas and 5 leiomyoma respectively.^[48]^ Although the sample size was small, it suggested that CH-EUS can accurately distinguish GISTs from benign lesions. In 2019, a retrospective analysis of 187 cases of ESELs shows that the sensitivity of CH-EUS for the diagnosis of esophageal stromal tumors and other subepithelial lesions was nearly 90%, and the specificity was 82%.^[49]^ This larger samples retrospective analysis provides a high reference value, suggesting that CH-EUS may be used as a reliable imaging technique for differentiating highly suspicious esophageal stromal tumor lesions in the future. After the diagnosis of esophageal stromal tumors, determining their malignant potential is equally important. Esophageal stromal tumors with malignant potential often have irregular blood vessels, which can be clearly shown on CH-EUS. One research comparatively analyzed 29 specimens of surgically resected esophageal lesions and their imaging performance under CH-EUS, finding that the sensitivity, specificity, and accuracy of CH-EUS in identifying irregular blood vessels and predicting GIST malignancy were 100%, 63%, and 83%, respectively.^[50]^ Another research retrospectively analyzed 143 patients, showing that the sensitivity of CH-EUS in diagnosing the malignant potential of GIST was as high as 96%, while its specificity was low at 53%.^[49]^ This suggests that CH-EUS not only has significant advantage in the diagnosis of esophageal stromal tumors, but also can more accurately diagnose malignant esophageal stromal tumors.

### 2.6. Esophageal leiomyosarcoma

Esophageal leiomyosarcoma is a rare malignant tumor of the esophagus, with an incidence of approximately 0.1% to 0.5% of all esophageal malignancies.^[44]^ Esophageal leiomyosarcoma usually originates from the muscularis propria of the esophagus. The appearance of esophageal leiomyosarcoma is similar to that of esophageal leiomyoma and esophageal stromal tumor on conventional imaging, but it has certain characteristics on EUS. EUS measures its malignant potential by measuring the lesion’s origin, maximum diameter, and the ratio of the long to short diameter of the lesion. It was found that esophageal leiomyosarcomas usually have a diameter >5 cm and a long/short diameter ratio >2.5 cm, indicating high malignancy.^[51]^ When it is challenging to make a definitive diagnosis by conventional EUS, obtaining smaller tumor tissues with EUS-FNA for cytological analysis can be a better diagnostic approach. In cases of esophageal leiomyosarcoma diagnosed by EUS-FNA, the lesions showed spindle cell features with cytological and nuclear pleomorphism after taking smears and were confirmed by postoperative pathology.^[52]^ This more definitive pathologic diagnosis can improve the preoperative diagnosis of esophageal leiomyosarcoma. At present, most preoperative diagnoses of esophageal leiomyosarcoma are involve tumors larger than 10 cm in diameter, with patients presenting more obvious symptoms of dysphagia. However, a case report showed that EUS-FNA could diagnose esophageal leiomyosarcoma with a diameter of about 4 cm.^[43]^ This suggests that EUS could be applied more broadly in the preoperative screening of patients with asymptomatic or small esophageal leiomyosarcoma.

### 2.7. Esophageal schwannoma (ES)

ES originates from Schwann cells of the nerve sheath and are usually benign lesions, with a 2% to 3% risk of malignancy.^[53,54]^ The GI tract is a rare site of schwannoma, accounting for <10%, and the esophagus has the lowest incidence of GI schwannoma.^[53,55]^ More than 70% of ES occurs in the lower esophagus.^[56]^ Under ordinary light microscope or CT, ES is often manifested as elevated lesions covering normal esophageal mucosal epithelium, which has no specific manifestations, so it is difficult to make an accurate diagnosis, while EUS can be more accurate in the diagnosis of schwannoma.^[57]^ On EUS, ES usually arises from the muscularis propria layer and show homogeneous or heterogeneous hypoechoic signals.^[55,58]^ One study has found that the accuracy of EUS in the diagnosis of ESs with diameter < 20 mm and diameter > 20 mm can be as high as 100% and 80%, respectively.^[59]^ Even ES with a diameter <20 mm can still have malignant potential. When EUS can only make a vague diagnosis, EUS-FNB can further confirm the pathological diagnosis by obtaining more tissue samples. When the conventional paraffin section cannot make a definite diagnosis, immunohistochemical examination can be performed by EUS-FNB to obtain a more accurate diagnostic rate.^[54]^

### 2.8. Esophageal neuroendocrine tumors (ENETs)

NETs are primary small-cell or large-cell esophageal carcinomas with positive neuroendocrine markers.^[60]^ ENETs are extremely rare, accounting for about 0.04% of all nets and 0.03% of all esophageal malignancies.^[61,62]^ ENETs usually occur in the lower esophagus, with dysphagia, weight loss and reflux esophagitis as the common symptoms.^[63]^ As the early symptoms of ENETs patients are not typical, the diagnosis is often advanced with lymph node metastasis, and the mid-term survival time of ENETS patients is only 4.2 to 18.5 months.^[64,65]^ According to the World Health Organization classification, the diagnosis of ENET currently relies on nuclear fission imaging and Ki-67 proliferation index to assess morphological differentiation (well/poorly differentiated) and cell proliferation potential (grade 1, 2 or 3) for tumor staging and subsequent treatment planning.^[66]^

The common imaging methods of ENET include EUS, CT, PET-CT and so on.^[61]^ EUS and CT both can be used to evaluate the regional lymph node metastasis and distant metastasis of ENET, but EUS is still the best imaging examination for the diagnosis of ENET. The accuracy of EUS in the diagnosis of ENET, especially small ENET, is significantly higher than that of CT.^[67,68]^ One study has shown that EUS may improve the detection rate of NET by about 25%.^[69]^ EUS-FNA or EUS-FNB can provide more accurate tissue sampling, accurate pathological examination and immunohistochemistry before surgery, and determine the tumor stage of ENET earlier to make more reasonable treatment plans. Another Study has shown that compared with postoperative pathological diagnosis results, preoperative EUS-FNA/FNB for NET patients to confirm the pathological diagnosis can make the accuracy rate more than 80%, which effectively avoids unnecessary surgical resection and reduces the occurrence of inadequate treatment.^[70]^ In addition, CH-EUS also plays a role in the diagnosis of ENETs. CH-EUS includes low enhancement, medium enhancement and high enhancement modes. One research has shown that the sensitivity, specificity and accuracy of low-enhanced CH-EUS in the diagnosis of NET are all more than 90%, which is beneficial to the vascular evaluation of ENET lesions and the diagnosis of smaller lesions.^[71]^ All these results suggest that the application of EUS and its combination technology is helpful for more accurate diagnosis and tumor staging of esophageal ENET patients.

## 3. EUS combined with other techniques in diagnosing ESELs

**Figure 2.**
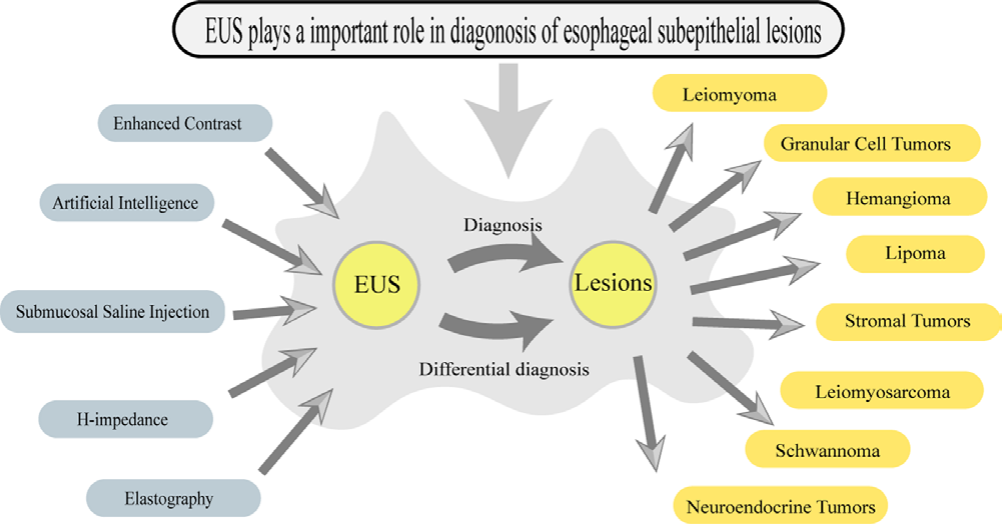
Characterization and application of common esophageal subepithelial tumors under EUS. EUS = endoscopic ultrasound.

In recent years, the combination of EUS with advanced techniques such as artificial intelligence (AI), submucosal saline injection (SSI), impedance measurement and contrast enhancement has become a new hotspot. Although the combined application of these technologies has not yet been widely used in clinical practice, it may play an auxiliary role in improving the diagnostic rate of EUS. The following section provide an overview of EUS (Fig. 2).

### 3.1. EUS and enhanced contrast

Compared with other imaging modalities, EUS still has limitations in evaluating tumor vascular characteristics. In recent years, CH-EUS has been increasingly used in, especially in the diagnosis of malignant lesions of liver,^[72]^ pancreas,^[73]^ bile duct^[74]^ and other areas.^[75]^ By injecting a contrast agent containing microbubbles into the vessel and observing the vibration of the microbubbles inside the vessel under EUS, CH-EUS has shown significant success in evaluating pancreatic tumor microperfusion, distinguishing pancreatic adenocarcinoma from NETs.^[76]^ Quantitative techniques of dynamic CH-EUS have also been recommended for perfusion imaging and monitoring anti-angiogenesis therapy.^[77]^ However, although it has been shown to improve the characterization of esophageal blood vessels, the application of CH-EUS in ESELs is not yet widespread. While the blood supply of ESELs is not abundant generally, the use of contrast agents can enhance the visibility of tumor boundaries and provide valuable information on tumor invasion depth.^[34]^ Studies have shown that GIST shows high enhancement under EUS, which are completely opposite to leiomyoma and lipoma which show low-enhanced, indicating the potential value of CH-EUS in differentiating benign and malignant ESELs.^[78]^ Therefore, CH-EUS is an effective method to distinguish benign submucosal tumor from GIST and to evaluate the malignant potential of GISTs. CH-EUS use more stable bubbles, which can achieve more accurate imaging effect, is superior to the conventional EUS in the diagnosis of ESELs.^[79]^ However, adequate B-ultrasound examination, otherwise CH-EUS is unlikely to provide additional information is necessary for this method to be effective. Contrast-enhanced EUS-guided fine needle aspiration (CE-EUS-FNA) can reduce the number of puncture required, thus lowering the risk of complications such as bleeding and infection.^[80]^

### 3.2. EUS and AI

EUS has certain advantages in determining the nature, size, origin, and depth of invasion of subepithelial lesions, and evaluating whether surrounding tissues and lymph nodes are invaded. However, the final diagnosis still relies on the subjective judgment of endoscopists.^[81]^ A review of 22 studies found that the overall missed diagnosis rate of gastric cancer by EUS was nearly 10%.^[82]^ This high misdiagnosis rate is likely due to the technical limitations of endoscopists, which prevent accurate identification of exposed lesions. Although EUS-FNA/FNB provides objective pathological results, these invasive procedures increase the risk of bleeding, perforation, obstruction, etc. Most importantly, the accuracy of EUS-FNA/FNB sampling also depends on the clinical skills of endoscopy physicians.

AI, primarily based on deep learning algorithms, provides EUS with an immediate output to explore all pixels of each image in all successive images from upper GI endoscopy without a lack of attention or fatigue.^[83]^ The application of AI in EUS effectively reduces the influence of subjective factors, increases the objectivity and accuracy of diagnostic results.^[84]^ At present, AI-based EUS diagnosis system has been widely used in the diagnosis of digestive tract lesions. A research retrospectively analyzed 16,110 images from 631 cases, developing an AI-based EUS diagnostic system that achieved an accuracy of 86.1% for 5 categories of digestive tract lesions, with significantly higher sensitivity and specificity than human endoscopists.^[85]^ A recent study collected 2431 patients containing more than 36,186 images as an overall dataset and found that EUS, with the assistance of AI, had a sensitivity and specificity of 92% and 82%, respectively, in distinguishing GIST from other esophageal diseases.^[86]^ Therefore, developing an AI-enhanced EUS diagnostic system for ESELs could significantly improve diagnostic outcomes. By collecting large sample data and employing deep learning algorithms, expert skills can be incorporated into real-time diagnostic processes.^[87]^ Interaction between AI and endoscopists can enhance the objectivity of diagnostic results, improve sensitivity and specificity, and thus reduce unnecessary diagnostic surgical resection.

### 3.3. EUS and SSI

SSI is a protective technique often used in endoscopic resection of ESELs to reduce injury by forming saline pads.^[88]^ SSI can also be used to improve the accuracy of EUS diagnosis. Because of the thin boundary between mucosa and submucosa, EUS is not satisfactory in distinguishing between mucosal and submucosal origin esophageal lesions, especially in early adenocarcinoma and atypical hyperplasia of the esophagus.^[89]^ When the depth of tumor invasion is difficult to determine, the mucosa and submucosa can be effectively separated by injecting normal saline under the mucosa, allowing EUS to clearly determine whether the tumor has infiltrated the submucosa.^[90]^

Previous studies have applied SSI combined with EUS increases the diagnostic accuracy for ESELs to 86.7%, which is nearly 27% higher than EUS alone.^[21]^ Recently, a study conducted an experiment with a larger sample and found that the accuracy of SSI combined with EUS for the diagnosis of ESELs increased by nearly 30% (up to 93.8%), effectively reducing unnecessary diagnostic endoscopic resection of esophagus.^[90]^ Another technique commonly used for depth of infiltration of esophageal lesions is magnifying endoscopy with narrow-band imaging, but SSI + EUS is more accurate for the diagnosis of ESELs compared to it.^[91]^

### 3.4. EUS and high-frequency impedance value

Endoscopic fine needle puncture/biopsy is the gold standard for the diagnosis of ESELs.^[7,11]^ Some researchers have determined the differential diagnosis of GISTs and non-GISTs by measuring the high-frequency impedance values (H-impedance) in GI stromal lesions and non-stromal lesions.^[92]^ The H-impedance of GISTs measured by EUS-FNB are significantly higher than those for leiomyoma, and the H-impedance values are significantly positively correlated with cell density. The accuracy rate and sensitivity of H-impedance in the differential diagnosis of GISTs and non-GISTs were 94.4% and 88.9%, and its diagnostic ability was not affected by lesion size.^[93]^ At present, this diagnostic method has not yet been widely developed in the diagnosis of ESELs. If EUS-FNB probe can be used to measure the H-impedance of ESELs smaller than 2 cm, it may have certain practical value in the identification and diagnosis of esophageal lesions by detecting H-impedance values of different lesions.

### 3.5. EUS and elastography

Elastography is a technique used to visualize tissue representations. During inflammation or tumor progression, the structure of normal tissues may change, and elastography is used to diagnose diseases by measuring compression-induced tissue deformation (strain) within a region of interest.^[94,95]^ Elastography has been used in ordinary ultrasound in the past. Elastography has recently been found to be useful in combination with EUS. Especially for GI lesions, ordinary imaging examination is limited to the surface of the lesion, while EUS-elastography can accurately distinguish the hardness of the lesion in the region of interest area, so as to diagnose or exclude the disease. One study has shown that when the EUS-elastography results of the pancreas indicate that the pancreas is significantly soft, pancreatic ductal adenocarcinoma can be basically excluded, and the negative predictive rate is 95%.^[95]^

On this basis, EUS-elastography is divided into qualitative and quantitative types.^[96]^ Qualitative elastography describes tissue stiffness in different colors, while quantitative elastography represents numerical results in strain ratio or hue histograms.^[97]^ For the comparison of pancreatic malignant tumors and inflammation, and the comparison of benign and malignant lymph nodes, quantitative analysis of EUS-elastography can be more intuitive to compare the histogram obtained by elastography, so as to obtain more objective comparison results.^[98]^ At present, EUS-elastography is mainly used for lymph node examination and N staging in esophageal lesions. Lymph nodes with a greater probability of malignancy appear blue on EUS-elastography for more than 50% of the surrounding area and other colors for the central area.^[99]^ Based on these characteristics, the accuracy and specificity of N staging of esophageal diseases are high, and the accuracy of some can be as high as 97%.^[99–101]^ In the future, EUS-elastography may play a similar role in the diagnosis of ESELs as it does in pancreatic diseases.

## 4. Discussion

ESELs, a common subset of esophageal abnormalities, primarily include leiomyoma, lipoma, hemangioma, and granulosa cell tumor. Small ESELs typically lack distinctive clinical manifestations and are often incidentally discovered during physical examinations or endoscopies. Larger leiomyomas and lipomas may protrude into the esophageal lumen, leading to symptoms such as dysphagia. Granulosa cell tumors, due to their malignant potential, are generally recommended for endoscopic or surgical resection. Hemangiomas, as a cause of chronic GI bleeding, warrant early intervention upon detection. Although most ESELs are benign, enhancing the clinical detection rate and accurate differential diagnosis facilitates early intervention and treatment, thereby improving patient prognosis.

EUS is crucial for diagnosing and differentiating ESELs. EUS allows for the visualization of the esophageal wall’s 5-layer structure, facilitating precise identification of the lesions’ origin, size, nature, and depth of infiltration. Additionally, EUS assesses invasion into surrounding tissues and lymph nodes. Various ESELs exhibit distinct characteristics under EUS, which can effectively differentiate these lesions based on layers, echo structures, and properties. Moreover, EUS-FNA and EUS-FNB enhance pathological diagnosis by obtaining small cell or tissue samples from the lesion site. Immunohistochemistry plays an important role when the pathology of esophageal submucosal lesions cannot be confirmed by conventional paraffin section. A multicenter study shows that EUS-FNB provides better tissue adequacy and integrity than FNA needles, leading to more accurate immunohistochemical diagnosis.^[102]^ In addition to simple tissue extraction, EUS-FNB can also be assisted by slow-pull, dry-suction, modified wet-suction to improve the integrity of tissue acquisition and improve the accuracy of pathological diagnosis.^[103]^ Studies have shown that the diagnostic rate of EUS-FNB without aspiration is significantly lower than that of other techniques, while modified wet-suction technique is significantly better than the dry aspiration technique in terms of sample tissue integrity, which can be considered as an improved means of EUS-FNB tissue extraction.^[104]^ EUS-FNB was superior to EUS-FNA. Perhaps in the future, EUS-FNB is likely to become the first-line imaging test for diagnosis of ESELs, with higher accuracy and sensitivity than EUS-FNA.^[59]^ The role of EUS in the diagnosis and differential diagnosis of several common ESELs, which are listed in order of frequency of occurrence. Consequently, clinical guidelines recommend EUS as a routine examination for ESELs.

In the past, the development and effectiveness of EUS were partly constrained by the experience and technical proficiency of endoscopists. While EUS-FNA/FNB improved the reliability of diagnostic results through pathological examination, it remained limited by the operator’s skill level and probe selection. However, the integration of EUS with other techniques can enhance diagnostic sensitivity and specificity, further reducing the rate of missed diagnoses. In recent years, EUS has increasingly been combined with advanced imaging, AI, and SSI, among other technologies, to improve its diagnostic capabilities.

Enhanced contrast imaging facilitates the visualization of the anatomical structure of esophageal vessels. The combined use of enhancement angiography and EUS allows for the detailed observation of subepithelial esophageal tumor boundaries and the depth of infiltration by highlighting blood vessels. AI-based EUS diagnostic systems can reduce the rates of missed and incorrect diagnoses that may result from the endoscopist’s experience, sampling techniques, work status, and other subjective factors. These systems, established through deep learning algorithms, offer higher diagnostic accuracy and objectivity. SSI can create a separation between the mucosal and submucosal layers, clarifying the anatomical structure, and plays a significant role in diagnosing the origin and depth of infiltration of subepithelial esophageal lesions via EUS. Measuring H-impedance under EUS and analyzing the electrophysiology of tumor cells can refine differential diagnosis and aid in detecting smaller subepithelial esophageal lesions. Endoscopic magnification narrow-band imaging is commonly used to assess the depth of tumor infiltration, and when combined with EUS, it enhances both the sensitivity and specificity of the diagnosis. This combined approach facilitates the diagnosis of the origin and depth of infiltration of esophageal lesions, providing a new method for the diagnosis and differential diagnosis of subepithelial esophageal lesions.

Although the combined application of these technologies is not been widely applied in clinical practice, they have provided innovative thinking for better application of EUS in the diagnosis of ESELs.

## Author contributions

**Conceptualization:** Wanwen Li, Bin He.

**Formal analysis:** Shichen Hu.

**Funding acquisition:** Bin He.

**Methodology:** Bin He.

**Supervision:** Mengqi Shao.

**Writing – original draft:** Wanwen Li, Mengqi Shao.

**Writing – review & editing:** Mengqi Shao, Shenglong Xie.

## References

[1] Sharzehi K Sethi A Savides T. AGA clinical practice update on management of subepithelial lesions encountered during routine endoscopy: expert review. Clin Gastroenterol Hepatol. 2022;20:2435–43.e4. 35842117 10.1016/j.cgh.2022.05.054

[2] Lewis RB Mehrotra AK Rodriguez P Levine MS. From the radiologic pathology archives: esophageal neoplasms: radiologic-pathologic correlation. Radiographics. 2013;33:1083–108. 23842973 10.1148/rg.334135027

[3] Lee LS Singhal S Brinster CJ. Current management of esophageal leiomyoma. J Am Coll Surg. 2004;198:136–46. 14698321 10.1016/j.jamcollsurg.2003.08.015

[4] Álvarez Martínez P Ramos Martínez PJ García González P Nieto-Jara A Del Caño Cerdán C García Riesco E. Primary esophageal diffuse large B-cell lymphoma. Rev Esp Enferm Dig. 2023;115:400–1. 36353960 10.17235/reed.2022.9298/2022

[5] Peño Muñoz L Boix Clemente C Martí Romero L. A rare endoscopic finding: primary esophageal melanoma. Rev Esp Enferm Dig. 2023;115:396–7. 36353961 10.17235/reed.2022.9244/2022

[6] Chen H Wang X Shao S Zhang J Tan X Chen W. Value of EUS in determining infiltration depth of early carcinoma and associated precancerous lesions in the upper gastrointestinal tract. Endosc Ultrasound. 2022;11:503–10. 36537388 10.4103/EUS-D-21-00218 PMC9921983

[7] Sooklal S Chahal P. Endoscopic ultrasound. Surg Clin North Am. 2020;100:1133–50. 33128884 10.1016/j.suc.2020.07.003

[8] Dawod E Nieto J Saab S. Endoscopic ultrasound-guided liver biopsy: where do we stand? Am J Gastroenterol. 2022;117:205–8. 34797223 10.14309/ajg.0000000000001551

[9] Tamura T Ashida R Kitano M. The usefulness of endoscopic ultrasound in the diagnosis of gallbladder lesions. Front Med (Lausanne). 2022;9:957557. 36106323 10.3389/fmed.2022.957557 PMC9465250

[10] Masuda S Koizumi K Shionoya K. Comprehensive review on endoscopic ultrasound-guided tissue acquisition techniques for solid pancreatic tumor. World J Gastroenterol. 2023;29:1863–74. 37032729 10.3748/wjg.v29.i12.1863 PMC10080698

[11] Deprez PH Moons LMG OʼToole D. Endoscopic management of subepithelial lesions including neuroendocrine neoplasms: European Society of Gastrointestinal Endoscopy (ESGE) Guideline. Endoscopy. 2022;54:412–29. 35180797 10.1055/a-1751-5742

[12] Kida M Kawaguchi Y Miyata E. Endoscopic ultrasonography diagnosis of subepithelial lesions. Dig Endosc. 2017;29:431–43. 28258621 10.1111/den.12854

[13] Kang S Kim DH Kim Y. Natural history of asymptomatic esophageal subepithelial tumors of 30 mm or less in size. J Korean Med Sci. 2022;37:e184. 35698837 10.3346/jkms.2022.37.e184 PMC9194489

[14] Yu Y Shen B Zhang C. Successful en bloc resection of large esophageal hemangioma by endoscopic submucosal dissection: a case report. Medicine (Baltimore). 2020;99:e22821. 33120807 10.1097/MD.0000000000022821 PMC7581152

[15] Ryu DG Kim SJ Choi CW. Combination conventional endoscopy and endoscopic ultrasound can differentiate between esophageal granular cell tumors and leiomyomas. Medicine (Baltimore). 2022;101:e31435. 36397402 10.1097/MD.0000000000031435 PMC9666135

[16] Aslanian HR Muniraj T Nagar A Parsons D. Endoscopic ultrasound in cancer staging. Gastrointest Endosc Clin N Am. 2024;34:37–49. 37973230 10.1016/j.giec.2023.09.009

[17] Chen C Song YL Wu ZY Chen J Zhang Y Chen L. Diagnostic value of conventional endoscopic ultrasound for lymph node metastasis in upper gastrointestinal neoplasia: a meta-analysis. World J Gastroenterol. 2023;29:4685–700. 37662859 10.3748/wjg.v29.i30.4685 PMC10472901

[18] Facciorusso A Crinò SF Fugazza A. Comparative diagnostic yield of different endoscopic techniques for tissue sampling of upper gastrointestinal subepithelial lesions: a network meta-analysis. Endoscopy. 2024;56:31–40. 37591258 10.1055/a-2156-0063

[19] Zhao Y Wang R Wang Y. Application of endoscopic ultrasound-guided-fine needle aspiration combined with cyst fluid analysis for the diagnosis of mediastinal cystic lesions. Thorac Cancer. 2019;10:156–62. 30480367 10.1111/1759-7714.12924 PMC6360264

[20] Joo DC Kim GH Lee MW Lee BE Baek DH Song GA. Diagnostic performance of endoscopic ultrasonography-guided fine-needle biopsy in upper gastrointestinal subepithelial tumors measuring 2–5 cm in size. Surg Endosc. 2022;36:8060–6. 35441867 10.1007/s00464-022-09243-5

[21] Osoegawa T Minoda Y Ihara E. Mucosal incision-assisted biopsy versus endoscopic ultrasound-guided fine-needle aspiration with a rapid on-site evaluation for gastric subepithelial lesions: a randomized cross-over study. Dig Endosc. 2019;31:413–21. 30723945 10.1111/den.13367

[22] De Moura DTH McCarty TR Jirapinyo P. EUS-guided fine-needle biopsy sampling versus FNA in the diagnosis of subepithelial lesions: a large multicenter study. Gastrointest Endosc. 2020;92:108–19.e3. 32105712 10.1016/j.gie.2020.02.021 PMC7340004

[23] Jiang W Rice TW Goldblum JR. Esophageal leiomyoma: experience from a single institution: esophageal leiomyoma. Dis Esophagus. 2013;26:167–74. 22458777 10.1111/j.1442-2050.2012.01345.x

[24] Sun LJ Chen X Dai YN. Endoscopic ultrasonography in the diagnosis and treatment strategy choice of esophageal leiomyoma. Clinics (Sao Paulo). 2017;72:197–201. 28492717 10.6061/clinics/2017(04)01 PMC5401615

[25] Huang YC Chiu NT Lu HI. FDG PET/CT and endoscopic ultrasound for preoperative T-staging of esophageal squamous cell carcinoma. Diagnostics (Basel). 2023;13:3083. 37835827 10.3390/diagnostics13193083 PMC10572619

[26] Shi YJ Yang X Yan S. Primary malignant melanoma of the esophagus: differentiation from esophageal squamous cell carcinoma and leiomyoma using dynamic contrast-enhanced CT findings. Abdom Radiol (NY). 2022;47:2747–59. 35668195 10.1007/s00261-022-03556-8 PMC9300547

[27] Xu G Qian J Chen M Ren G Chen H. Endoscopic ultrasonography for the diagnosis and selecting treatment of esophageal leiomyoma. J Gastroenterol Hepatol. 2012;27:521–5. 21916996 10.1111/j.1440-1746.2011.06929.x

[28] Jiang T Yu J Chen L. Clinical value of endoscopic ultrasonography for esophageal leiomyoma in elder patients. J Vis Surg. 2017;3:137–137. 29078697 10.21037/jovs.2017.09.03 PMC5639007

[29] Codipilly DC Fang H Alexander JA Katzka DA Ravi K. Subepithelial esophageal tumors: a single-center review of resected and surveilled lesions. Gastrointest Endosc. 2018;87:370–7. 28782509 10.1016/j.gie.2017.07.043

[30] Kang H Kim SJ Young Do M. Endoscopic ultrasound-guided fine needle aspiration and biopsy for cytohistological diagnosis of gallbladder cancer: a multicenter retrospective study. Gastrointest Endosc. 2024:S0016510724001834.

[31] Pan W Wu J Liu C He Y Yang J. Esophageal low-grade intraepithelial neoplasia overlying multiple leiomyomas: a case report and review of the literature. Front Oncol. 2022;12:994005. 36387267 10.3389/fonc.2022.994005 PMC9659895

[32] Ryu DG Choi CW Kim SJ. Clinical outcomes of esophageal granular cell tumors with different endoscopic resection methods. Sci Rep. 2023;13:10738. 37400629 10.1038/s41598-023-37998-x PMC10317998

[33] Shi Y Chai N Zhong L. Experience with esophageal granular cell tumors: clinical and endoscopic analysis of 22 cases. Dig Dis Sci. 2021;66:1233–9. 32474763 10.1007/s10620-020-06337-9

[34] Reddy NK Ioncică AM Săftoiu A Vilmann P Bhutani MS. Contrast-enhanced endoscopic ultrasonography. World J Gastroenterol. 2011;17:42–8. 21218082 10.3748/wjg.v17.i1.42 PMC3016678

[35] Gao ZY Liu XB Pandey S. Clinicopathological features of esophageal schwannomas in mainland China: systematic review of the literature. Int J Clin Oncol. 2021;26:284–95. 33216242 10.1007/s10147-020-01809-4

[36] Stašek M Aujeský R Škarda J. Malignant granular cell tumor of the esophagus: a case report. Ann Thorac Cardiovasc Surg. 2020;26:359–64. 33012751 10.5761/atcs.cr.20-00117 PMC7801172

[37] Iwamuro M Tanaka T Kanzaki H Kawano S Kawahara Y Okada H. Esophageal granular cell tumors can be differentiated from leiomyomas using endoscopic ultrasonography. Intern Med. 2018;57:1509–15. 29321437 10.2169/internalmedicine.9816-17 PMC6028673

[38] Zhongsheng L Yan D Ezzat R. Endoscopic submucosal dissection: a safe and effective alternative to surgical intervention for esophageal hemangioma. Surg Laparosc Endosc Percutan Tech. 2024;34:124–8. 38372527 10.1097/SLE.0000000000001266

[39] Gralnek IM Camus Duboc M Garcia-Pagan JC. Endoscopic diagnosis and management of esophagogastric variceal hemorrhage: European Society of Gastrointestinal Endoscopy (ESGE) Guideline. Endoscopy. 2022;54:1094–120. 36174643 10.1055/a-1939-4887

[40] Al Shamousi K AL-Naamani Z Abbas Q Al-Busafi S El Bingawi H. Treatment with endoscopic ultrasound (EUS)-guided transesophageal coil embolization without sclerotherapy: a novel therapy for giant distal esophageal hemangioma. Cureus. 2022. 10.7759/cureus.25303 PMC9236642 35774648

[41] Yang D Duckworth L Draganov P. More than meets the eye: esophageal invasive mucinous adenocarcinoma masquerading as a submucosal lipoma on endoscopic ultrasound. Endoscopy. 2014;46(S 01):E502–3. 25314219 10.1055/s-0034-1377764

[42] Faulx AL Kothari S Acosta RD. The role of endoscopy in subepithelial lesions of the GI tract. Gastrointest Endosc. 2017;85:1117–32. 28385194 10.1016/j.gie.2017.02.022

[43] Ebi M Sakamoto K Inoue S. Esophageal leiomyosarcoma diagnosed by endoscopic ultrasound-guided fine-needle aspiration biopsy and cured with surgical resection. Intern Med. 2019;58:2479–83. 31118374 10.2169/internalmedicine.2219-18 PMC6761329

[44] Rocco G Trastek VF Deschamps C Allen MS Miller DL Pairolero PC. Leiomyosarcoma of the esophagus: results of surgical treatment. Ann Thorac Surg. 1998;66:894–6; discussion 897. 9768947 10.1016/s0003-4975(98)00684-5

[45] Parab TM DeRogatis MJ Boaz AM. Gastrointestinal stromal tumors: a comprehensive review. J Gastrointest Oncol. 2018;10:144–54. 10.21037/jgo.2018.08.20 PMC6351301 30788170

[46] Moisini I Amin K Mallery S Stewart J Mettler T. Efficacy of endoscopic-guided fine-needle aspiration in the diagnosis of gastrointestinal spindle cell tumors. Diagn Cytopathol. 2018;46:663–9. 31012545 10.1002/dc.23976

[47] Trindade AJ Benias PC Alshelleh M. Fine-needle biopsy is superior to fine-needle aspiration of suspected gastrointestinal stromal tumors: a large multicenter study. Endosc Int Open. 2019;07:E931–6. 10.1055/a-0953-1640 PMC6624113 31304239

[48] Kannengiesser K Mahlke R Petersen F. Contrast-enhanced harmonic endoscopic ultrasound is able to discriminate benign submucosal lesions from gastrointestinal stromal tumors. Scand J Gastroenterol. 2012;47:1515–20. 23148660 10.3109/00365521.2012.729082

[49] Tang JY Tao KG Zhang LY. Value of contrast-enhanced harmonic endoscopic ultrasonography in differentiating between gastrointestinal stromal tumors: a meta-analysis. J Dig Dis. 2019;20:127–34. 30714350 10.1111/1751-2980.12710

[50] Sakamoto H Kitano M Matsui S. Estimation of malignant potential of GI stromal tumors by contrast-enhanced harmonic EUS (with videos). Gastrointest Endosc. 2011;73:227–37. 21295636 10.1016/j.gie.2010.10.011

[51] Serrano C George S. Leiomyosarcoma. Hematol Oncol Clin North Am. 2013;27:957–74. 24093170 10.1016/j.hoc.2013.07.002

[52] Stelow EB Jones DR Shami VM. Esophageal leiomyosarcoma diagnosed by endoscopic ultrasound-guided fine-needle aspiration. Diagn Cytopathol. 2007;35:167–70. 17415921 10.1002/dc.20606

[53] Fukushima N Aoki H Fukazawa N Ogawa M Yoshida K Yanaga K. Schwannoma of the small intestine. Case Rep Gastroenterol. 2019;13:294–8. 31341461 10.1159/000501065 PMC6639570

[54] Tomizawa K Miyazaki T Yamaguchi A. Malignant peripheral nerve sheath tumour of the oesophagus: a case report. Surg Case Rep. 2020;6:186. 32737607 10.1186/s40792-020-00954-2 PMC7394979

[55] Jiao J Fan X Luo L. Efficacy of endoscopic ultrasound and endoscopic resection for esophageal schwannoma. Scand J Gastroenterol. 2023;58:963–9. 36880341 10.1080/00365521.2023.2185867

[56] Li B Wang X Zou WL Yu SX Chen Y Xu HW. Endoscopic resection of benign esophageal schwannoma: three case reports and review of literature. 2020;8:5690–700 10.12998/wjcc.v8.i22.5690 PMC7716328 33344562

[57] Shimamura Y Winer S Marcon N. A schwannoma of the distal esophagus. Clin Gastroenterol Hepatol. 2016;14:A19–20. 10.1016/j.cgh.2016.07.023 27475621

[58] Crinò S Bernardoni L Manfrin E Parisi A Gabbrielli A. Endoscopic ultrasound features of pancreatic schwannoma. Endosc Ultrasound. 2016;5:396. 28000633 10.4103/2303-9027.195873 PMC5206830

[59] Sekine M Miura T Fujiwara J. Utility of endoscopic ultrasonography-guided fine-needle biopsy (EUS-FNB) for diagnosing small subepithelial lesions (<20 mm). J Ultrasound. 2022;25:35–40. 33511507 10.1007/s40477-020-00548-6 PMC8964910

[60] Wu IC Chu YY Wang YK. Clinicopathological features and outcome of esophageal neuroendocrine tumor: a retrospective multicenter survey by the digestive endoscopy society of Taiwan. J Formos Med Assoc. 2021;120:508–14. 32600867 10.1016/j.jfma.2020.06.024

[61] Lee CG Lim YJ Park SJ. The clinical features and treatment modality of esophageal neuroendocrine tumors: a multicenter study in Korea. BMC Cancer. 2014;14:569. 25098730 10.1186/1471-2407-14-569 PMC4133602

[62] Babu Kanakasetty G Dasappa L Lakshmaiah KC. Clinicopathological profile of pure neuroendocrine neoplasms of the esophagus: a South Indian center experience. J Oncol. 2016;2016:1–6. 10.1155/2016/2402417 PMC4906204 27340404

[63] Poston LM Gupta S Alvarado CE. Contemporary outcomes of esophageal and gastroesophageal junction neuroendocrine tumors. Dis Esophagus. 2023;36:doad001. 36688874 10.1093/dote/doad001

[64] Yoshinami Y Nishimura E Hosokai T. Rare malignant neoplasm of the esophagus: current status and future perspectives. Jpn J Clin Oncol. 2024;54:111–20. 37861097 10.1093/jjco/hyad144 PMC10849183

[65] Pantvaidya GH Pramesh CS Deshpande MS Jambhekar NA Sharma S Deshpande RK. Small cell carcinoma of the esophagus: the Tata Memorial Hospital experience. Ann Thorac Surg. 2002;74:1924–7. 12643374 10.1016/s0003-4975(02)04061-4

[66] Assarzadegan N Montgomery E. What is new in the 2019 World Health Organization (WHO) classification of tumors of the digestive system. Arch Pathol Lab Med. 2019;145:664–77. 10.5858/arpa.2019-0665-RA PMC9281538 32233993

[67] Paiella S Landoni L Rota R. Endoscopic ultrasound-guided fine-needle aspiration for the diagnosis and grading of pancreatic neuroendocrine tumors: a retrospective analysis of 110 cases. Endoscopy. 2020;52:988–94. 32498099 10.1055/a-1180-8614

[68] Angelis CD Pellicano R Rizzetto M Repici A. Role of endoscopy in the management of gastroenteropancreatic neuroendocrine tumours. Minerva Gastroenterol Dietol. 2011;57. 21587152

[69] Frilling A Akerström G Falconi M. Neuroendocrine tumor disease: an evolving landscape. Endocr Relat Cancer. 2012;19:R163–85. 22645227 10.1530/ERC-12-0024

[70] Tacelli M Bina N Crinò SF. Reliability of grading preoperative pancreatic neuroendocrine tumors on EUS specimens: a systematic review with meta-analysis of aggregate and individual data. Gastrointest Endosc. 2022;96:898–908.e23. 35863518 10.1016/j.gie.2022.07.014

[71] Franchellucci G Andreozzi M Carrara S. Contrast enhanced EUS for predicting solid pancreatic neuroendocrine tumor grade and aggressiveness. Diagnostics (Basel). 2023;13:239. 36673049 10.3390/diagnostics13020239 PMC9857765

[72] Otsuka Y Kamata K Hyodo T. Utility of contrast-enhanced harmonic endoscopic ultrasonography for T-staging of patients with extrahepatic bile duct cancer. Surg Endosc. 2022;36:3254–60. 34462868 10.1007/s00464-021-08637-1

[73] Möller K Jenssen C Braden B. Comments on and Illustrations of the EFSUMB CEUS guidelines: transabdominal and endoscopic ultrasound features of intrapancreatic metastases and the role of multiparametric imaging and EUS-guided sampling in rare pancreatic tumors. Cancers. 2023;15:2546. 37174015 10.3390/cancers15092546 PMC10177255

[74] Takahashi K Ozawa E Shimakura A Mori T Miyaaki H Nakao K. Recent advances in endoscopic ultrasound for gallbladder disease diagnosis. Diagnostics (Basel). 2024;14:374. 38396413 10.3390/diagnostics14040374 PMC10887964

[75] Wu J Zhuang M Zhou Y Zhan X Xie W. The value of contrast-enhanced harmonic endoscopic ultrasound in differential diagnosis and evaluation of malignant risk of gastrointestinal stromal tumors (<50 mm). Scand J Gastroenterol. 2023;58:542–8. 36369879 10.1080/00365521.2022.2144437

[76] Kataoka K Ishikawa T Ohno E. Differentiation between solid pseudopapillary neoplasm of the pancreas and nonfunctional pancreatic neuroendocrine neoplasm using endoscopic ultrasound. Pancreas. 2022;51:106–11. 35195603 10.1097/MPA.0000000000001966

[77] Alvarez-Sanchez M Gincul R Lefort C Napoleon B. Role of contrast-enhanced harmonic endoscopic ultrasound in submucosal tumors. Endosc Ultrasound. 2016;5:363. 28000626 10.4103/2303-9027.190928 PMC5206823

[78] Pesenti C Bories E Caillol F. Characterization of subepithelial lesions of the stomach and esophagus by contrast-enhanced EUS: a retrospective study. Endosc Ultrasound. 2019;8:43–9. 30264741 10.4103/eus.eus_89_17 PMC6400084

[79] Dietrich C. Controversies in EUS. Endosc Ultrasound. 2021;10:1. 33586695 10.4103/EUS-D-21-00024 PMC7980683

[80] Lai JH Lin CC Lin HH Chen MJ. Is contrast-enhanced endoscopic ultrasound-guided fine needle biopsy better than conventional fine needle biopsy? A retrospective study in a medical center. Surg Endosc. 2022;36:6138–43. 35484412 10.1007/s00464-022-09253-3 PMC9283143

[81] Lau LHS Sung JJY. Treatment of upper gastrointestinal bleeding in 2020: new techniques and outcomes. Dig Endosc. 2021;33:83–94. 32216134 10.1111/den.13674

[82] Sharma P Hassan C. Artificial intelligence and deep learning for upper gastrointestinal neoplasia. Gastroenterology. 2022;162:1056–66. 34902362 10.1053/j.gastro.2021.11.040

[83] Chen TH Kuo CF Lee C Yeh T-S Lan J Huang S-C. Artificial intelligence model for a distinction between early-stage gastric cancer invasive depth T1a and T1b. J Cancer. 2024;15:3085–94. 38706899 10.7150/jca.94772 PMC11064248

[84] Yuan XL Liu W Lin YX. Effect of an artificial intelligence-assisted system on endoscopic diagnosis of superficial oesophageal squamous cell carcinoma and precancerous lesions: a multicentre, tandem, double-blind, randomised controlled trial. Lancet Gastroenterol Hepatol. 2024;9:34–44. 37952555 10.1016/S2468-1253(23)00276-5

[85] Hirai K Kuwahara T Furukawa K. Artificial intelligence-based diagnosis of upper gastrointestinal subepithelial lesions on endoscopic ultrasonography images. Gastric Cancer. 2022;25:382–91. 34783924 10.1007/s10120-021-01261-x

[86] Zhang B Zhu F Li P Zhu J. Artificial intelligence-assisted endoscopic ultrasound in the diagnosis of gastrointestinal stromal tumors: a meta-analysis. Surg Endosc. 2023;37:1649–57. 36100781 10.1007/s00464-022-09597-w

[87] Huang J Fan X Liu W. Applications and prospects of artificial intelligence-assisted endoscopic ultrasound in digestive system diseases. Diagnostics (Basel). 2023;13:2815. 37685350 10.3390/diagnostics13172815 PMC10487217

[88] Mou Y Ye L Qin X. Impact of submucosal saline injection during cold snare polypectomy for colorectal polyps sized 3–9 mm: a multicenter randomized controlled trial. Am J Gastroenterol. 2023;118:1848–54. 37207320 10.14309/ajg.0000000000002329

[89] Young PE Gentry AB Acosta RD Greenwald BD Riddle M. Endoscopic ultrasound does not accurately stage early adenocarcinoma or high-grade dysplasia of the esophagus. Clin Gastroenterol Hepatol. 2010;8:1037–41. 20831900 10.1016/j.cgh.2010.08.020

[90] He LJ Xie C Wang ZX. Submucosal saline injection followed by endoscopic ultrasound versus endoscopic ultrasound only for distinguishing between T1a and T1b esophageal cancer. Clin Cancer Res. 2020;26:384–90. 31615934 10.1158/1078-0432.CCR-19-1722

[91] Lee MW Kim GH I H. Predicting the invasion depth of esophageal squamous cell carcinoma: comparison of endoscopic ultrasonography and magnifying endoscopy. Scand J Gastroenterol. 2014;49:853–61. 24957951 10.3109/00365521.2014.915052

[92] Poggi L Bernui GM Romani DA Gavidia AF Poggi LA. Persistent and De Novo GERD after sleeve gastrectomy: manometric and pH-impedance study findings. Obes Surg. 2023;33:87–93. 36394780 10.1007/s11695-022-06126-5

[93] Minoda Y Esaki M Ihara E. Auxiliary diagnosis of subepithelial lesions by impedance measurement during EUS-guided fine-needle biopsy. Gastrointest Endosc. 2023;97:977–84. 36460086 10.1016/j.gie.2022.11.022

[94] Dietrich C Bibby E Jenssen C Saftoiu A Iglesias-Garcia J Havre R. EUS elastography: how to do it? Endosc Ultrasound. 2018;7:20. 29451165 10.4103/eus.eus_49_17 PMC5838723

[95] Dietrich CF Cantisani V. Current status and perspectives of elastography. Eur J Radiol. 2014;83:403–4. 23540945 10.1016/j.ejrad.2013.02.028

[96] Iglesias-Garcia J De La Iglesia-Garcia D Lariño-Noia J Dominguez-Muñoz JE. Endoscopic ultrasound (EUS) guided elastography. Diagnostics (Basel,). 2023;13:1686. 37238170 10.3390/diagnostics13101686 PMC10217724

[97] Iglesias-Garcia J Lindkvist B Lariño-Noia J Domínguez-Muñoz JE; Sample Organization. Endoscopic ultrasound elastography. Endosc Ultrasound. 2012;1:8–16. 24949330 10.7178/eus.01.003 PMC4062202

[98] Dietrich C Hirche T Ott M Ignee A. Real-time tissue elastography in the diagnosis of autoimmune pancreatitis. Endoscopy. 2009;41:718–20. 19618344 10.1055/s-0029-1214866

[99] Paterson S Duthie F Stanley AJ. Endoscopic ultrasound-guided elastography in the nodal staging of oesophageal cancer. World J Gastroenterol. 2012;18:889–95. 22408347 10.3748/wjg.v18.i9.889 PMC3297047

[100] Knabe M Günter E Ell C Pech O. Can EUS elastography improve lymph node staging in esophageal cancer? Surg Endosc. 2013;27:1196–202. 23093233 10.1007/s00464-012-2575-y

[101] Sazuka T Akai T Uesato M. Assessment for diagnosis of lymph node metastasis in esophageal cancer using endoscopic ultrasound elastography. Esophagus. 2016;13:254–63. 27429608 10.1007/s10388-016-0521-0 PMC4923115

[102] Zhao Y Xiong D Aruna, . Fine needle biopsy versus fine needle aspiration in the diagnosis of immunohistochemistry-required lesions: a multicenter study with prospective evaluation. Endosc Ultrasound. 2023;12:456–64. 38948128 10.1097/eus.0000000000000028 PMC11213591

[103] Crinò SF Conti Bellocchi MC Di Mitri R. Wet-suction versus slow-pull technique for endoscopic ultrasound-guided fine-needle biopsy: a multicenter, randomized, crossover trial. Endoscopy. 2023;55:225–34. 35915956 10.1055/a-1915-1812

[104] Facciorusso A Crinò SF Ramai D. Comparative diagnostic performance of different techniques for EUS-guided fine-needle biopsy sampling of solid pancreatic masses: a network meta-analysis. Gastrointest Endosc. 2023;97:839–48.e5. 36657607 10.1016/j.gie.2023.01.024

